# Myocardial steatosis across the spectrum of human health and disease

**DOI:** 10.1113/EP091566

**Published:** 2023-12-08

**Authors:** Andrew P. Oneglia, Lidia S. Szczepaniak, Vlad G. Zaha, Michael D. Nelson

**Affiliations:** ^1^ Applied Physiology and Advanced Imaging Laboratory, Department of Kinesiology, College of Nursing and Health Innovation University of Texas at Arlington Arlington Texas USA; ^2^ Biomedical Consulting in MRS Albuquerque New Mexico USA; ^3^ Division of Cardiology, Internal Medicine University of Texas Southwestern Medical Center Dallas Texas USA; ^4^ Advanced Imaging Research Center University of Texas Southwestern Medical Center Arlington Texas USA; ^5^ Clinical Imaging Research Center University of Texas at Arlington Arlington Texas USA; ^6^ Center for Healthy Living and Longevity University of Texas at Arlington Arlington Texas USA

**Keywords:** magnetic resonance, myocardial triglyceride content, steatosis

## Abstract

Preclinical data strongly suggest that myocardial steatosis leads to adverse cardiac remodelling and left ventricular dysfunction. Using ^1^H cardiac magnetic resonance spectroscopy, similar observations have been made across the spectrum of health and disease. The purpose of this brief review is to summarize these recent observations. We provide a brief overview of the determinants of myocardial triglyceride accumulation, summarize the current evidence that myocardial steatosis contributes to cardiac dysfunction, and identify opportunities for further research.

## Abstract (highlights)

- **What is the topic of this review?**
  The role of myocardial steatosis on cardiac morphology and function.
- **What advances does it highlight?**
  Myocardial steatosis contributes to adverse cardiac remodelling and left ventricular dysfunction.

## Graphical abstract

Preclinical data strongly suggest that myocardial steatosis leads to adverse cardiac remodelling and ventricular dysfunction. Evidence is reviewed of clinical translation using ^1^H magnetic resonance spectroscopy. Figure created using BioRender.com.

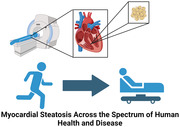

## 1 INTRODUCTION

More than two decades have passed since the seminal discovery that intracellular triglyceride accumulation within the hearts (myocardial steatosis) of obese Zucker Diabetic Fatty rats leads to adverse cardiac remodelling and left ventricular dysfunction (Zhou et al., 2000), via lipotoxic pathways (Goldberg et al., 2012; Schulze et al., 2016; Wende & Abel, 2010). Clinical translation of this important work was largely limited to clinical biopsies and post‐mortem analyses (Anderson et al., 2009; Björnson et al., 2020; Gizurarson et al., 2015; Knapp et al., 2020; Liu et al., 2012; Mazzali et al., 2015), but greatly expanded following the advent of ^1^H cardiac magnetic resonance spectroscopy (den Bakermans et al., 2021; Hollander et al., 1994; Reingold et al., 2005; Szczepaniak et al., 2003), as previously reviewed (J. M. McGavock et al., 2006; Szczepaniak et al., 2007). Since these early clinical observations, the field has continued to evolve, with myocardial steatosis now described across the spectrum of health and disease. This brief review summarizes these later observations while identifying remaining knowledge gaps and opportunities for future research.

## 2 MYOCARDIAL TRIGLYCERIDE CONTENT AS A MASS (IM)BALANCE

**FIGURE 1:**
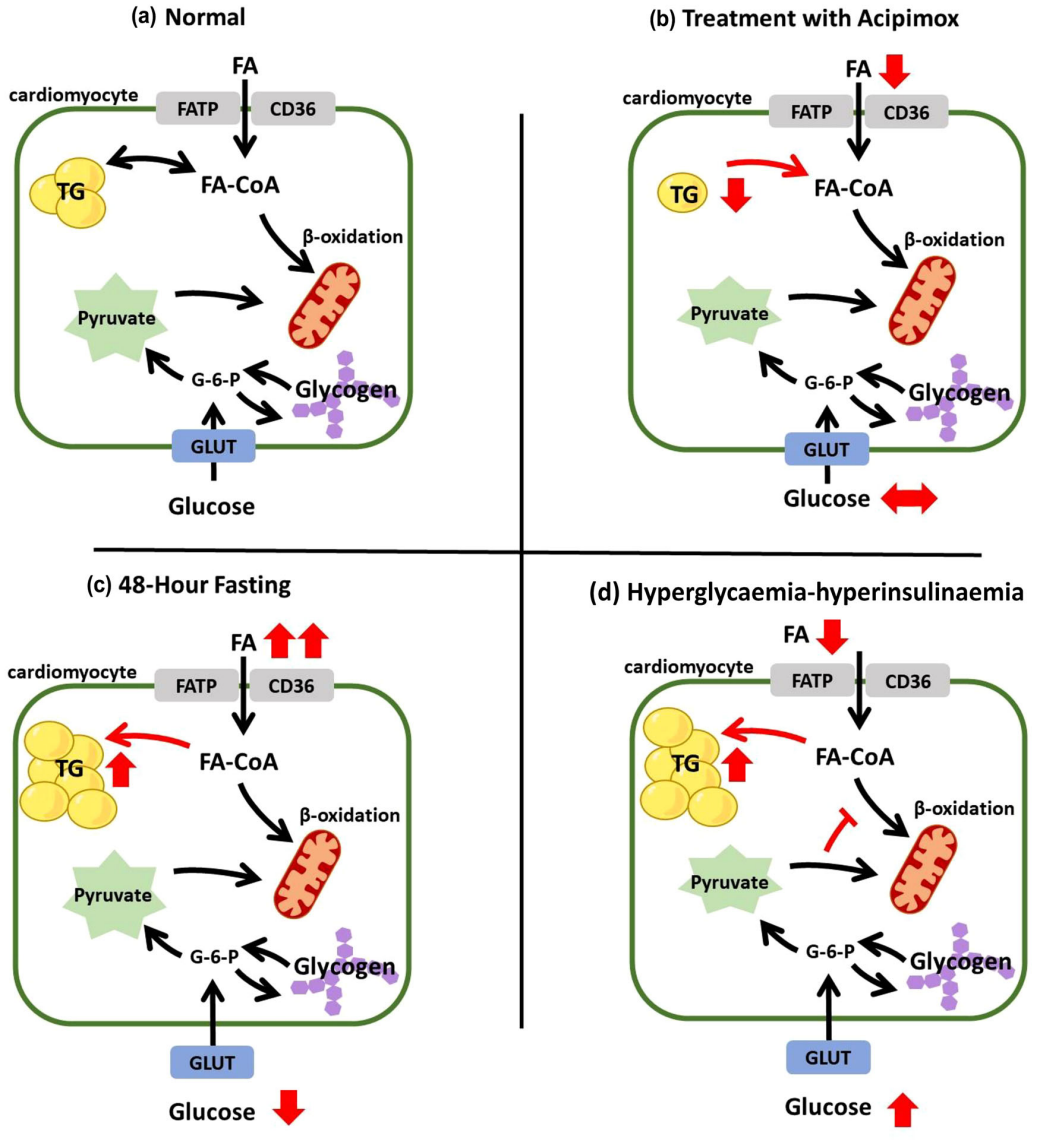
Schematic representation of a cardiomyocyte under normal conditions (a), during systemic treatment with acipimox (b, a lipid lowering agent), during 48‐h fasting (c), and in response to a whole‐body hyperglycaemic–hyperinsulinaemic clamp (d). FA, free fatty acids; G‐6‐P, glucose‐6‐phosphate; GLUT, glucose transporter; TG, triglyceride.

Myocardial triglyceride content is ultimately determined by the balance between substrate availability and substrate utilization (Figure 1). Under fasting conditions (Figure 1a), the majority of energy is derived from the oxidation of free fatty acids, with a limited pool of stored triglyceride. Indeed, data from 10 separate studies in young, normal weight, healthy adults suggest myocardial triglyceride content is approximately 0.5% fat/water (Gaborit, Kober, et al., 2012; Hammer, van der Meer, Lamb, Schar et al., 2008; Oneglia et al., 2023; Petritsch et al., 2016; Sai et al., 2013, 2015; Smajis et al., 2020; van der Meer et al., 2007, van der Meer, Hammer et al., 2008; Wolf et al., 2021). Moreover, myocardial triglyceride content is relatively stable within the small variations in substrate availability/utilization that may occur with day‐to‐day changes in dietary or exercise habits (Aengevaeren et al., 2020; Bucher et al., 2014; Ith et al., 2014; Smajis et al., 2020; van der Meer, Hammer et al., 2008). However, it is possible to shift the balance between lipid oxidation and storage, such that myocardial triglyceride content can change, even in healthy adults. For example, reducing circulating free fatty acids with acipimox (an anti‐lipolytic agent) reduces myocardial triglyceride content (Figure 1b) (Lehto et al., 2012; Winhofer et al., 2015). In contrast, multiple lines of evidence highlight acute increases in myocardial triglyceride content in the healthy myocardium when substrate availability exceeds utilization (Figure 1c,d), such as during periods of severe caloric restriction and fasting (Hammer, van der Meer, Lamb, Schar et al., 2008; Oneglia et al., 2023; van der Meer et al., 2007), following aerobic exercise in a fasted state (Bilet et al., 2011), and during hyperinsulinaemia–hyperglycaemia (Winhofer et al., 2012). Among these latter examples, substrate availability exceeds utilization in clear and close relation to nutritional state. Indeed, low nutrient intake depletes glycogen stores so that the body must instead rely on free fatty acids and ketones for energy, both of which increase their circulating concentrations via lipolysis of adipose tissue. The effect on myocardial triglyceride content can be seen in as little as 4 h post‐exercise and 48‐h of fasting (Bilet et al., 2011; Oneglia et al., 2023).

## 3 AGE AND SEX

Several studies suggest that myocardial triglyceride content increases with age (Petritsch et al., 2015; Sarma et al., 2013; van der Meer, Rijzewijk et al., 2008); however, the total sample size available to support this conclusion is relatively small, with very few reports measuring myocardial triglyceride content in individuals >65 years of age. Sex does not appear to influence myocardial triglyceride content in young, normal weight, healthy adults (Oneglia et al., 2023; Petritsch et al., 2016; Winhofer et al., 2012); however, the interaction between sex and metabolic dysfunction/disease has not been thoroughly investigated. Where available, sex as a biological variable is described in each of the following sections.

## 4 OBESITY AND DIABETES

**FIGURE 2:**
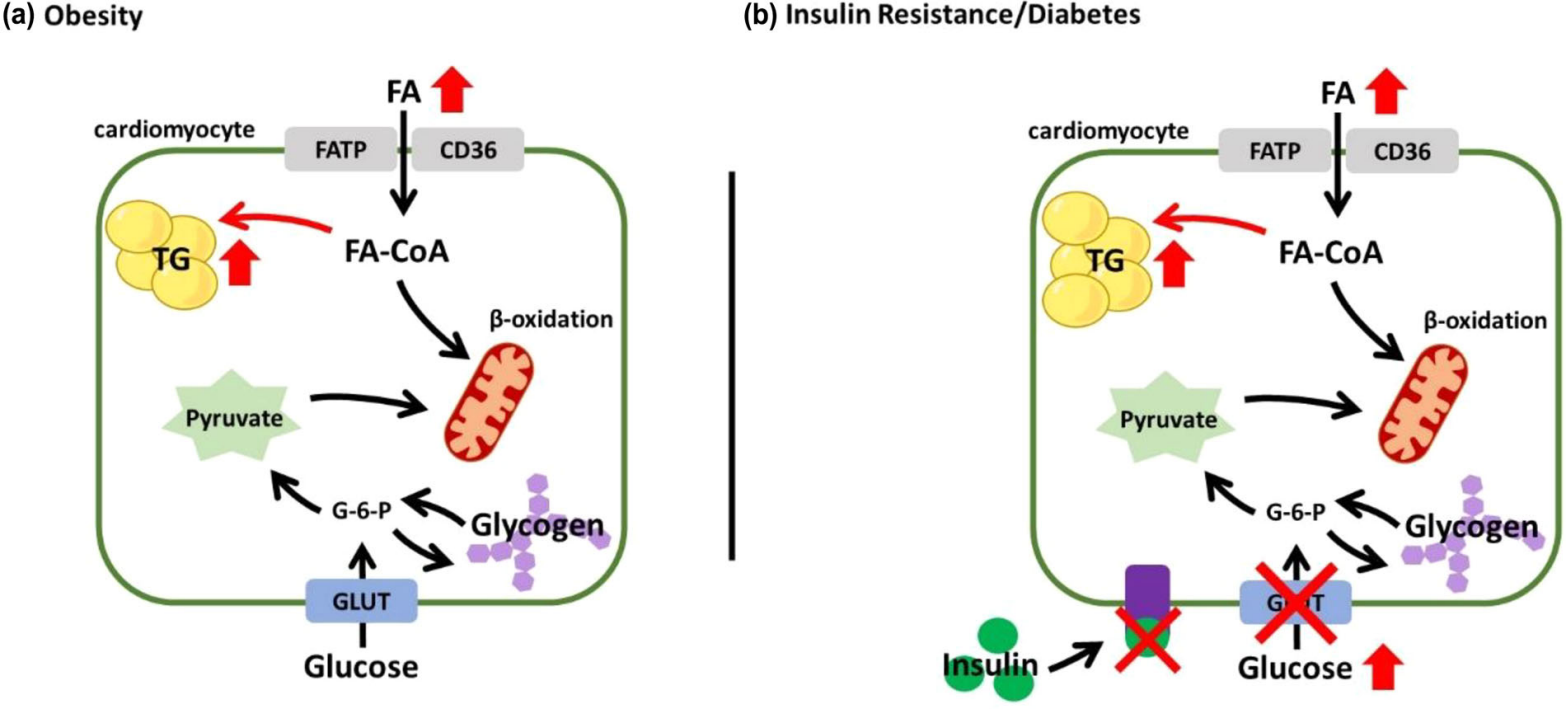
Schematic representation of a cardiomyocyte during obesity (a) and insulin resistance/diabetes (b). CD36, fatty acid translocase; FA, free fatty acids; FATP, fatty acid transport protein; G‐6‐P, glucose‐6‐phosphate; GLUT, glucose transporter; TG, triglyceride.

Mounting evidence support a stepwise increase in myocardial triglyceride content with obesity, impaired glucose tolerance, and type 2 diabetes (T2D) (Dong et al., 2023; Gaborit, Kober, et al., 2012; Iozzo et al., 2009; J. M. McGavock et al., 2007). Our interpretation of this stepwise increase is illustrated in Figure 2, where obesity‐mediated increases in myocardial triglyceride content are largely explained by an associated increase in substrate availability (Figure 2a) (Banerjee et al., 2015; Gaborit, Kober et al., 2012; Hannukainen et al., 2018; Lai et al., 2015; Liu et al., 2014; Rayner et al., 2018, 2019). This effect appears to occur in both adults and adolescent boys (Banerjee et al., 2015). However, obesity is rarely defined by a simple increase in circulating free fatty acids, but rather also involves varying degrees of insulin resistance. Therefore, abnormal substrate utilization, in addition to an obesity‐related increase in substrate availability, augments myocardial triglyceride content among those at risk of developing T2D and those with diagnosed T2D (Figure 2b) (Gao et al., 2020; Graner et al., 2013; Levelt, Mahmod et al., 2016, Levelt, Pavlides et al., 2016; Muniyappa et al., 2015; Nyman et al., 2013; Rijzewijk et al., 2008).

While myocardial triglyceride content may be associated with ectopic fat deposition elsewhere in the body, including visceral adiposity (Gaborit, Kober et al., 2012; Graner et al., 2013; Iozzo et al., 2009; Liu et al., 2014; Rayner et al., 2018), pericardial fat content (Gaborit, Kober et al., 2012; Graner et al., 2013; Iozzo et al., 2009) and hepatic steatosis (Graner et al., 2013; Liu et al., 2014), these associations are not perfect, emphasizing the importance of measuring myocardial triglyceride content directly. Results from interventional trials further demonstrate this point, as summarized in Table 1. This is perhaps best exemplified following bariatric surgery, where myocardial triglyceride content remains elevated 6 months after surgery, despite a ∼20% reduction in body mass, and marked reductions in pericardial, visceral and subcutaneous fat (Gaborit, Jacquier et al., 2012; Hannukainen et al., 2018; van Schinkel et al., 2014). We interpret this dissociative pattern to reflect a shift in myocardial substrate utilization, whereby improvements in myocardial glucose utilization, without changes in myocardial fatty acid utilization, result in a preservation of myocardial triglyceride content. Myocardial steatosis decreases only when fatty acid utilization exceeds fatty acid supply, so that the heart may instead rely on stored triglycerides. This could also explain why the majority of the T2D literature finds no direct relationship between myocardial steatosis and whole body insulin sensitivity/resistance (Graner et al., 2013; Iozzo et al., 2009; Kosi‐Trebotic et al., 2017; Krssak et al., 2011; Muniyappa et al., 2015; Winhofer, Krssak et al., 2014), and a number of glucose‐lowering drug trials have improved glycaemic control without altering myocardial triglyceride content (Bizino et al., 2020; Dutour et al., 2016; Gaborit et al., 2021; Hiruma et al., 2021; Hsu et al., 2019; J. McGavock et al., 2012; Paiman et al., 2020; van der Meer et al., 2009).

**TABLE 1:** Myocardial triglyceride content response to interventional trials in obesity and diabetes mellitus.

Reference	Subjects (m/f)	Age (years)	Study design	Medications	Results
Exercise intervention					
Schrauwen‐Hinderling et al. (2010)	14 overweight to obese subjects (14/—)	Mean: 58 (SEM: 1)	12‐week, combination aerobic and resistance, training programme	N/A	• mTG ↓ mean 45%, but weight and body fat % ↔, after exercise training
Schrauwen‐Hinderling et al. (2011)	11 T2D patients (11/—)	Mean: 60 (SEM: 1)	12‐week, combination aerobic and resistance, training programme	Oral glucose‐lowering, lipid‐lowering, anti‐hypertensive and/or blood diluent agents were continued	• mTG, weight, body fat %, and total body fat all ↔ after exercise training
Honkala et al. (2017)	28 healthy vs. 16 IGT subjects, 13 of which were T2D patients (44/—)	Healthy range: 40–55 IGT range: 43–53	Randomized, 2‐week HIIT or MICT	Oral glucose‐lowering agents were continued	• mTG tends to ↓ with HIIT, but not MICT, irrespective of glucose tolerance • Epi‐ and pericardial fat ↓ in both exercise conditions, irrespective of glucose tolerance
Dietary Intervention					
Utz et al. (2013)	38 overweight and obese subjects (—/38)	Mean: 43 (SD: 9)	Randomized, 6‐month reduced‐CHO or reduced‐fat hypocaloric dietary interventions	N/A	• mTG ↓ mean 25%, along with a ↓ in weight and total fat mass, irrespective of diet type
Andersson et al. (2016)	68 overweight to obese subjects (—/68)	Palaeolithic diet Mean: 60 (SD: 6) Nordic diet Mean: 60 (SD: 6)	Randomized, 24‐month ad libitum Palaeolithic or Nordic Nutrition Recommendation dietary interventions	N/A	• mTG ↔, but weight ↓, 6 and 24 months after diet initiation, irrespective of diet type
Hammer, van der Meer, Lamb, de Boer et al. (2008)	11 T2D patients (11/—)	Mean: 58 (SD: 5)	3‐day regular diet vs. randomized VLCD and VLCD + acipimox treatment	Metformin was continued	• mTG ↑ mean 48% after VLCD, but ↔ after VLCD + acipimox • weight ↓, but HTG ↔, in both VLCD conditions
Hammer, Snel et al. (2008)	12 T2D patients (7/5)	Mean: 48 (SEM: 3)	16‐week VLCD	Glucose lowering therapy was discontinued	• mTG ↓ mean 27%, along with a ↓ in weight and HTG
Jonker et al. (2014)	14 T2D patients with obstructive CAD and/or myocardial perfusion defects (7/7)	Mean: 57 (SEM: 3)	regular diet vs. 3‐day VLCD	Glucose‐lowering therapy was adjusted to maintain comparable glucose levels between study days	• mTG ↑ mean 33% after VLCD, but weight ↓ while VAT, SAT, HTG and pericardial fat ↔
Airhart et al. (2016)	16 T2D patients (4/12)	MCFA diet Mean: 48 (SEM: 3) LCFA diet Mean: 52 (SEM: 3)	Double‐blind, randomized 2‐week MCFA or LCFA rich eucaloric dietary intervention	Medication use was continued, but no subject was taking insulin	• mTG, waist/hip ratio, and HTG ↔, but weight ↓, irrespective of diet type
Other					
Jankovic et al. (2012)	10 T2D patients (6/4)	Mean: 58 (SEM: 3)	10 days’ standardized insulin therapy	Metformin, lipid‐lowering and/or anti‐hypertensive agents were continued	• mTG ↑ mean 80%
Hammer, Jonker et al. (2008)	10 T1D patients (5/5)	Mean: 41 (SEM: 3)	Randomized, euglycaemic and 24‐h hyperglycaemic conditions	Hyperglycaemic condition was achieved by reducing basal and bolus insulin infusions ∼50%	• mTG ↔ after hyperglycaemia compared to euglycaemia
Abdesselam et al. (2015)	21 morbidly obese subjects (4/17)	Mean: 42 (SD: 2)	32‐month follow‐up visit after bariatric surgery	N/A	• mTG ↓ 32 months post‐surgery, whereas VAT, SAT, EAT, HTG, PTG all ↓ 6 months post‐surgery
Wolf et al. (2016)	8 T2D patients (6/2)	Mean: 56 (SD: 11)	6‐h randomized, placebo‐controlled acipimox treatment	Glucose‐lowering therapy and statin therapy were omitted	• mTG ↓ mean 41% after acipimox treatment • mTG ↔ after placebo treatment

Abbreviations: CAD, coronary artery disease; CVD, cardiovascular disease; EAT, epicardial adipose tissue; HIIT, high‐intensity interval training; HTG, hepatic triglyceride content; IGT, impaired glucose tolerance; LCFA, long‐chain fatty acid; MCFA, medium‐chain fatty acid; MICT, moderate‐intensity continuous training; mTG, myocardial triglyceride content; N/A, not available; PTG, pancreatic triglyceride content; SAT, subcutaneous adipose tissue; T1D, type‐1 diabetes mellitus; T2D, type‐2 diabetes mellitus; VAT, visceral adipose tissue; VLCD, very‐low calorie diet.

In a relatively small sample, Iozzo et al. (2009) first reported that myocardial triglyceride content was lower in obese women than men but was comparable between sexes with impaired glucose tolerance or T2D, suggesting that women may be protected from obesity‐related increases in myocardial triglyceride content. Studies conducted since, however, suggest otherwise (Dong et al., 2023; Gaborit, Kober et al., 2012). Indeed, myocardial triglyceride content is still elevated in overweight and obese women compared to normal weight and overweight men (Liu et al., 2014). Furthermore, sex does not influence the independent relationship between obesity and myocardial steatosis among adults across a range of obesity (Banerjee et al., 2015). As such, sex does not appear to influence myocardial triglyceride content in obesity.

The functional consequence of myocardial triglyceride accumulation in obesity and T2D remains incompletely understood. Although multiple cross‐sectional studies have identified an independent association between myocardial steatosis and cardiac function (Banerjee et al., 2015; Dong et al., 2023; Gao et al., 2020; Korosoglou et al., 2012; Levelt, Mahmod et al., 2016; Ng et al., 2010; Rijzewijk et al., 2008) and/or left ventricular (LV) concentric remodelling (Banerjee et al., 2015; Jankovic et al., 2012; Jonker et al., 2014; Levelt, Mahmod et al., 2016), improvements in cardiac function do not always follow reductions in myocardial triglyceride content and vice versa (van der Meer et al., 2009; Zib et al., 2007). These latter observations have contributed to the understanding that cardiac function is not related to myocardial steatosis per se, but rather the accumulation of toxic intermediates from lipid metabolism (Schulze et al., 2016; Wende & Abel, 2010; Goldberg et al., 2012).

## 5 HEART DISEASE

Myocardial triglyceride content varies across different forms of heart disease (Graner et al., 2014; Nakae et al., 2010; Sai et al., 2017). Myocardial ischaemia appears to promote the accumulation of triglycerides, likely related to a metabolic switch away from free fatty acid oxidation. Indeed, myocardial triglyceride content is elevated in patients with ischaemic coronary artery disease (CAD), but not patients with non‐ischaemic CAD or non‐CAD controls (Hannukainen et al., 2016). Moreover, myocardial steatosis was elevated in women with ischaemia but non‐obstructed coronary arteries, and inversely related to LV diastolic function (Wei et al., 2016). Notably, in this latter cohort, triglyceride content was not elevated elsewhere in the body (Hannukainen et al., 2016; Wei et al., 2016), arguing against systemic metabolic disease and in support of the ischaemia hypothesis. Myocardial triglyceride content was also elevated in heart failure with preserved ejection fraction (Mahmod et al., 2018; Wu et al., 2020), but not heart failure with reduced ejection fraction (Wu et al., 2020) or end‐stage heart failure (Chokshi et al., 2012). However, these latter two cohorts were primarily non‐obese and non‐diabetic, which may have influenced the results (Sharma et al., 2004).

Interventional studies aimed at regressing myocardial steatosis in heart disease are limited. In one investigation, 1 year of high‐intensity exercise training, without dietary intervention, promoted positive cardiac and vascular remodelling in adults at risk for heart failure, but did not affect myocardial steatosis (Hearon et al., 2022). Other investigations targeted myocardial lipid composition in heart failure, rather than total content per se, but research in this area is extremely limited (Chang et al., 2020; Liao et al., 2016). Hata et al. 2022 are the only group to investigate the longitudinal effects of myocardial steatosis on LV diastolic function. Using computed tomography, patients with no or mild coronary artery stenosis were grouped by the presence or absence of myocardial fat deposition at baseline. Echocardiography was then performed at baseline, 1–2 years and 2–3 years after the baseline assessment. When compared to patients without excess myocardial fat deposition, those with excess myocardial fat deposition had worse LV diastolic function at baseline and follow‐up. Moreover, the decline in LV diastolic function with ageing appeared accelerated in patients with excess myocardial fat deposition. While these data support the negative effect of myocardial steatosis on LV function, a direct cause‐and‐effect relationship between the two is limited by the study's retrospective design. Therefore, future prospective, longitudinal studies are needed to confirm these results.

## 6 HUMAN IMMUNODEFICIENCY VIRUS

The influence of human immunodeficiency virus (HIV) and associated highly active anti‐retroviral therapy has received considerable attention with respect to myocardial steatosis and its influence of future cardiovascular disease risk. Indeed, myocardial triglyceride content is often elevated among individuals with HIV, and associated with worsening LV systolic and diastolic function (Holloway et al., 2013; Nelson et al., 2014; Shitole et al., 2023; Thiara et al., 2015; Toribio et al., 2019). Several mechanisms may contribute to an increase in myocardial triglyceride content with HIV, including age (Chew et al., 2017; Neilan et al., 2020; Toribio et al., 2019), body mass index (Lai et al., 2017; Neilan et al., 2020), ectopic body fat deposition (Chew et al., 2017; Diaz‐Zamudio et al., 2015; Howard et al., 2016; Thiara et al., 2015) and even duration of anti‐retroviral therapy (Lai et al., 2017; Nelson et al., 2014). Sex does not appear to influence this association, with myocardial triglyceride content elevated in both men and women with HIV. Studies aimed at regressing myocardial triglyceride content in HIV are actively underway (NCT02344290) (Grinspoon et al., 2019).

## 7 SPECIAL POPULATIONS

Investigations into other clinical populations not yet described are summarized in Table 2, ranging from valvular heart disease to metabolic disease (e.g., non‐alcoholic fatty liver disease, generalized lipodystrophy, Cushing's syndrome). Where correlation analyses were performed, the accumulation of myocardial steatosis is often associated with adverse ventricular remodelling and/or cardiac dysfunction, except in non‐alcoholic fatty liver disease.

**TABLE 2:** Myocardial triglyceride content in special populations.

References	Clinical population	Results
Mahmod et al. (2013)	39 aortic stenosis patients vs. 20 matched controls	• mTG ↑ in severe aortic stenosis • mTG ↓ in patients after aortic valve replacement to concentrations similar to controls
Graner et al. (2015)	75 non‐alcoholic fatty liver disease patients divided into tertiles according to hepatic TG	• mTG ↑ with ↑ adiposity (BMI, hepatic TG, VAT, SAT, epi‐ and pericardial fat)
Brittain et al. (2016)	6 pulmonary arterial hypertension vs. 8 controls	• mTG ↑ in PAH • Despite higher FFA availability, PAH experience impaired FA utilization so that lipotoxicity ensued
Gizurarson et al. (2015)	34 patients with atrial fibrillation vs. 17 controls	• mTG ↓ in atrial fibrillation when assessed directly from atrial biopsies
Nelson et al. (2013)	6 generalized lipodystrophy patients vs. 6 matched controls	• mTG ↑ in generalized lipodystrophy with concurrent concentric LV hypertrophy
Wolf et al. (2021)	23 Cushing's syndrome patients vs. 27 matched controls	• mTG ↔ despite greater epi‐ and pericardial fat mass in patients • mTG ↔ after treatment and remission
Scherer et al. (2014)	10 hypothyroidism patients vs. 10 matched controls	• mTG ↔ • After levothyroxine treatment, mTG ↓ with concurrent improvements in LV morphology and filling dynamics
Winhofer, Wolf et al. (2014)	10 acromegaly patients vs. 10 matched controls	• mTG and hepatic TG ↔ but pericardial fat ↓ in acromegaly • After treatment, mTG and hepatic TG ↔ but pericardial fat ↑
Wolf et al. (2014)	8 familial hypocalcuric hypercalcaemia patients vs. 9 matched controls	• mTG, hepatic TG, VAT, and SAT ↔
Knottnerus et al. (2020)	14 patients with long‐chain fatty acid β oxidation disorders vs. 14 matched controls	• mTG ↔
Gastl et al. (2019)	11 cardiac amyloidosis patients vs. 11 matched controls	• mTG ↓ in cardiac amyloidosis

BMI, body mass index; FA, fatty acid; FFA, free fatty acid; mTG, myocardial triglyceride content; PAH, pulmonary arterial hypertension; SAT, subcutaneous adipose tissue; TG, triglyceride; VAT, visceral adipose tissue.

## 8 CONCLUSION

Myocardial triglyceride content is determined by the (im)balance between free fatty acid substrate availability and utilization. Myocardial steatosis increases with obesity and insulin‐resistance, is elevated in multiple disease states, and may be an important source of cardiac dysfunction and/or adverse remodelling. More work is still needed, however, to address remaining gaps in the literature, including the influence of age and sex, and the independent role myocardial steatosis plays in the development and progression of heart disease.

## AUTHOR CONTRIBUTIONS

Conception or design of the work: Andrew P. Oneglia, Lidia S. Szczepaniak, Vlad G. Zaha, Michael D. Nelson . Drafting the work or revising it critically for important intellectual content: Andrew P. Oneglia, Lidia S. Szczepaniak, Vlad G. Zaha, Michael D. Nelson. All authors have read and approved the final version of this manuscript and agree to be accountable for all aspects of the work in ensuring that questions related to the accuracy or integrity of any part of the work are appropriately investigated and resolved. All persons designated as authors qualify for authorship, and all those who qualify for authorship are listed.

## CONFLICT OF INTEREST

The authors declare no conflicts of interest.
